# A Novel RNA Virus Related to Sobemoviruses Confers Hypovirulence on the Phytopathogenic Fungus *Sclerotinia sclerotiorum*

**DOI:** 10.3390/v11080759

**Published:** 2019-08-16

**Authors:** Ayesha Azhar, Fan Mu, Huang Huang, Jiasen Cheng, Yanping Fu, Muhammad Rizwan Hamid, Daohong Jiang, Jiatao Xie

**Affiliations:** 1 State Key Laboratory of Agricultural Microbiology, Hubei Key Laboratory of Plant Pathology, Huazhong Agricultural University, Wuhan 430070, Hubei Province, China; 2 Provincial Key Laboratory of Plant Pathology of Hubei Province, College of Plant Science and Technology, Huazhong Agricultural University, Wuhan 430070, China

**Keywords:** *Sclerotinia sclerotiorum*, mycovirus, plant virus, *Sobemovirus*

## Abstract

Infection by diverse mycoviruses is a common phenomenon in *Sclerotinia sclerotiorum*. In this study, the full genome of a single-stranded RNA mycovirus, tentatively named Hubei sclerotinia RNA virus 1 (HuSRV1), was determined in the hypovirulent strain 277 of *S. sclerotiorum*. The HuSRV1 genome is 4492 nucleotides (nt) long and lacks a poly (A) tail at the 3ˊ- terminus. Sequence analyses showed that the HuSRV1 genome contains four putative open reading frames (ORFs). ORF1a was presumed to encode a protein with a conserved protease domain and a transmembrane domain. This protein is 27% identical to the P2a protein encoded by the subterranean clover mottle virus. ORF1b encodes a protein containing a conserved RNA-dependent RNA polymerase (RdRp) domain, which may be translated into a fusion protein by a -1 ribosome frameshift. This protein is 45.9% identical to P2b encoded by the sowbane mosaic virus. ORF2 was found to encode a putative coat protein, which shares 23% identical to the coat protein encoded by the olive mild mosaic virus. ORF3 was presumed to encode a putative protein with an unknown function. Evolutionary relation analyses indicated that HuSRV1 is related to members within *Sobemovirus*, but forms a unique phylogenetic branch, suggesting that HuSRV1 represents a new member within *Solemoviridae*. HuSRV1 virions, approximately 30 nm in diameter, were purified from strain 277. The purified virions were successfully introduced into virulent strain Ep-1PNA367, resulting in a new hypovirulent strain, which confirmed that HuSRV1 confers hypovirulence on *S. sclerotiorum*.

## 1. Introduction

Destructive crop diseases are caused by plant pathogenic fungi globally, making disease control for sustainable agriculture a constant and costly challenge [1,2]. Fungicides are widely used to control fungal diseases, but a large number of crops pose a difficulty with obtaining disease-resistant cultivars, and the increased application of fungicides also carries a serious threat to human health and the environment. There is, therefore, a dire need to develop novel methods that reduce the use of fungicides for fungal disease control. Some mycoviruses are natural promising agents known to control fungal diseases through the induction of hypovirulence [3,4,5]. They are prevalent in all major groups of phytopathogenic fungi [3]. Most of the known double-stranded RNA (dsRNA) viruses yield symptomless infections in their host [3]. However, a number of cases are reported in which mycoviruses significantly alleviate a disease by inducing hypovirulence or enhance disease symptoms by inducing hypervirulence [3,4,5,6], and therefore mycoviruses associated hypovirulence are employed as biological control agents against fungal diseases [3,4,5,6]. Mycoviruses utilize various routes of transmission, including through hyphae, spores, fungal cell wall division, and insect vector transmission (Lycoriella ingenua) [6,7]. Mycoviruses can cause the abnormal growth rate of the hosts, affect the production of sclerotia, and decrease or abrogate the pathogen’s virulence, thereby providing a safe method for plant disease control [8].

*Sclerotinia sclerotiorum* (Lib.) de Bary is a globally distributed ascomycetous phytopathogenic fungus with a wide host range of over 400 plant species, including sunflower (*Helianthus annuus*), rapeseed (*Brassica napus)*, *Arabidopsis thaliana* (research model plant), and soybean (*Glycine max*) [9], thereby causing significant yield losses [10]. Due to lack of more sophisticated control methods, cultivation practices almost always rely on the application of fungicides to control *S. sclerotiorum*-induced diseases. Nonetheless, continuous fungicide spraying often leads to the development of fungicide resistance [11,12,13]. Mycoviruses that confer hypovirulence have the potential to develop virocontrol agents to manage Sclerotinia disease [5,14,15]. dsRNA elements in *S. sclerotiorum* and its associated hypovirulence phenomenon were first reported in the 1990s [16]. Later, a rich diversity of mycoviruses were detected from *S. sclerotiorum* and successfully characterized at the molecular level, including single-stranded RNA (ssRNA) viruses (hypovirus, sclerodarnavirus, mitovirus, sclerotimonavirus, and seven unclassified ssRNA viruses), dsRNA viruses (partitivirus, megabirnavirus, mycoreovirus, botybirnavirus), and an single-stranded DNA (ssDNA) virus (gemycircularvirus) [5,17,18,19,20,21]. Most of these viruses reduce the growth and virulence of *S. sclerotiorum*. *S. sclerotiorum*, therefore, represents an excellent system for exploring virus-host interactions and for the discovery of novel approaches to control fungal disease.

In this study, we noted that hypovirulent *S. sclerotiorum* strain 277 was co-infected with two unique viruses: the mycotymovirus, Sclerotinia sclerotiorum mycotymovirus-like 1 (SsMTV1), and a new (+) ssRNA mycovirus designated as Hubei sclerotinia RNA virus 1 (HuSRV1). SsMTV1 is identical to the previously reported mycovirus Fusarium graminearum mycotymovirus 1 (FgMTV1) [22], and HuSRV1 is related to, but significantly different from sobemoviruses. Accordingly, we mainly conducted genome characterization to elucidate the molecular features of HuSRV1, investigate its impact on virulence, and to understand its biological properties in *S. sclerotiorum*.

## 2. Materials and Methods 

### 2.1. Strains and Culture Conditions

*S. sclerotiorum* strain 277 was isolated from a sclerotium obtained from a diseased rapeseed (*B. napus*) plant in the Hubei Province, China. *S. sclerotiorum* strain Ep-1PNA367 was a single-ascospore isolate of strain Ep-1PN, and has normal colony morphology and strong virulence on its hosts [23]. Strain Ep-1PNA367R was labeled with the hygromycin-resistance gene (*hygromycin B phosphotransferase*). The biological properties of strain Ep-1PNA367R are not significantly different from those of its parent strain Ep-1PNA367. All *S. sclerotiorum* strains were cultured on potato dextrose agar (PDA) at 20–22 °C and stored on PDA slants at 4 °C.

### 2.2. Comparison of Biological Characteristics

To compare growth rates, mycelial agar plugs from actively growing colony margins of strain Ep-1PNA367R, 277, and their derivatives (strains A367R-HuSRV1, A367-SsMTV1) were transferred onto PDA plates (9 cm in diameter) and then incubated at 20 °C. The colony diameter of each strain was measured at 24 and 48 h post-inoculation (hpi). The colony morphology of each strain was examined daily until mature sclerotia were produced. To assess the difference in virulence among strains Ep-1PNA367R, 277 and its derivatives, actively growing mycelial agar plugs (0.5 cm in diameter) were inoculated onto detached leaves of rapeseed, and the inoculated host leaves were placed in an incubator at 20 °C and 100 % relative humidity. The development of diseased lesions on the rapeseed leaves was examined and photographed at 48 hpi. Each treatment involved more than three replicates. Biological properties data were analyzed by one-way analysis of variance using SAS^®^8.0 program. Differences with *p* < 0.01 were considered statistically significant.

### 2.3. Purification and Observation of Virions

The virions were isolated from strain 277 or A367R-HuSRV1, following the method described by Xiao et al. [19], with minor modifications. Briefly, the strains were grown at 20 °C for 5–6 days on sterilized cellophane films placed on PDA or in potato dextrose broth (PDB). Approximately 30 g of mycelia were harvested by straining through Miracloth in a Buchner funnel. The mycelia were then ground in buffer A (0.1 M sodium phosphate buffer at pH 7.0 containing 1 g of dithiothreitol) and the mixture was gently shaken on ice for 30 min. The resulting homogenate was separated by high-speed centrifugation (12,000 rpm for 30 min), and the supernatant was collected for virions extraction by ultracentrifugation. After a round of high-speed centrifugation (26,000 rpm for 2 h in the Beckman SW28 rotor, Beckman Instruments, Inc., Fullerton, CA, USA), virion pellets were re-suspended in buffer A overnight at 4 °C. Virions were then purified by centrifugation through a layer of 36% (*wt/wt*) CsCl in buffer A for 6 h at 36,000 rpm in the Beckman SW41 rotor. The purified virions (the light-absorbing band) were collected by puncturing the centrifuge tube with a needle and syringe. The harvested virion samples were diluted with buffer A (ratio 1:1) and centrifuged at 36,000 rpm for 2 h in the Beckman SW41 rotor. The final pellets were suspended in buffer A overnight. The virions were then stained with 2 % (*w/v*) phosphotungstic acid solution (pH 7.4), and examined under a transmission electron microscope (Model Tecnai G2, 200kV, FEI Company, Hillsboro, OR, USA).

### 2.4. Nucleic Acid Extraction, Metatranscriptomic Sequencing, and Full-Length cDNA Cloning

Fungal total RNA samples were extracted using RNAiso, according to the RNA extraction reference (Takara, Dalian, China), and sent to Shanghai Biotechnology Corporation (Shanghai, China) for metatranscriptomic sequencing based on the method of the depleted rRNA with Ribo-Zero™ rRNA Removal Kit (Illumina, San Diego, CA, USA). To obtain clean sequences, the raw reads from metatranscriptomic sequencing were processed to remove the adaptor sequences, discard low-quality reads, and any read with any number of ambiguous base calls. These clean transcripts were first matched against the genome sequences of *S. sclerotiorum* using the Bowtie (1.0) software. The unmatched RNAs were next assembled into longer contiguous sequences (contigs) in the Velvet software before they were employed in searches in Non-redundant protein sequences (nr) of GenBank database (http://www.ncbi.nlm.nih.gov/) in BlastX program using the Diamond software (version 0.9.25) under default parameters with the exception of the e-value lower e^−5^. The contigs that were identical or complementary to the viral genomic sequence were extracted and identified as potential viral sequences.

Viral genome RNA samples from virions were extracted using the phenol/chloroform extraction method [19]. RNA samples from virions were used for cDNA cloning and terminal determination as previously described [24] with minor modification. An anchor primer PC3-T7 loop (5′-p-GGATCCCGGGAATTCGGTAATACGACTCACTATATTTTTATAGTGAGTCGTATTA-OH-3′) was ligated to purified ssRNA using T4 RNA ligase and used for the RT reaction. The primer PC2 (5′-CCGAATTCCCGGGATCC-3′) designed based on the corresponding sequence of the PC3-T7 loop, and sequence-specific primers designed based on the available sequence and proximal regions sequences were used for the amplification of terminal sequences. The expected PCR products were recovered and purified with a gel extraction kit (Axygen, New York, USA), and then cloned into the pMD18-T vector (TaKaRa, Dalian, China) for sequencing. To achieve high-quality consensus sequences, and to avoid laboratory PCR artifacts, each nucleotide of full-length cDNA was sequenced for a minimum of three times.

### 2.5. Sequence and Phylogenetic Analyses

The basic features of the complete genome of mycovirus were analyzed using DNAMAN software. Potential ORFs and conserved domain(s) were identified using ORF finder and CD-search on the website of the National Center for Biotechnology Information (NCBI) (http://www.ncbi.nlm.nih.gov) and motifs scan website (http://www.genome.jp/tools/motif/). The virus-encoded amino acid sequence was analyzed with the TMHMM server (version 2.0, http://www.cbs.dtu.dk/services/TMHMM/) to predict potential transmembrane domains.

Sequence alignment was carried out using the M-Coffee web server (http://tcf_dev.vital-it.ch/apps/tcoffee/play?name=mcoffee). The maximum likelihood (ML) trees were constructed with PhyML 3.1 using the best-fit model (LG+G+I+F) selected by ProtTest350, with subtree pruning and regrafting (SPR) algorithms and 16 categories of gamma-distributed substitution rates. The reliability of internal branches was evaluated with SPR supports. The sequences of previously reported viruses and other reference sequences in this study were retrieved from the NCBI GenBank database (http://www.ncbi.nlm.nih.gov/genomes) and were employed for comparative analyses. Prediction of RNA pseudoknots was performed using the program DotKnot [25] as implemented at http://dotknot.csse.uwa.edu.au. The free energy value for each predicted structure was provided by the respective prediction program, and the structural image of RNA pseudoknot was visualized using program VARNAv3-7 [26] on the webserver (http://varna.lri.fr). The Phyre2 server was used to predict the three-dimensional structure of protein sequences with a homology modeling method [27]. The structural images of proteins were created using the PyMOL molecular viewer.

### 2.6. Protoplast Preparation and Transfection

Strain Ep-1PNA367 was served as the recipient strain. Protoplast preparation and transfection with the purified virions were performed as previously described [28]. Purified virions were mixed with protoplast (10^6^~10^7^/mL) of strain Ep-1PNA367 in the presence of polyethylene glycol (PEG) 4000 and then spreading of this solution on regeneration medium (RM) plates. RM media plates were then incubated at 20–22 °C for seven days. Ten clones were randomly picked from each plate and then grown on PDA media plates overloading with the cellophane membrane for three days. Total RNA of each new clone was used for cDNA preparation and for RT-PCR with virus-specific primers.

### 2.7. Horizontal Transmission of Hypovirulence Traits

To assess the potential horizontal transmission of hypovirulence traits of strain 277, dual culturing of strains Ep-1PNA367R and 277 on PDA plates was carried out to allow for contact between the two colonies as a previously described method [28]. After the contact was achieved, mycelia agar plugs from colony margins of strain Ep-1PNA367R were placed onto a fresh PDA plate containing 25 mg/mL hygromycin; only the strain (such as strain Ep-1PNA367R or viruses-infected strain Ep-1PNA367R-V) labeled with the hygromycin-resistance gene was expected to grow. Mycelia plugs were picked up from the new colony from PDA containing hygromycin and transferred onto fresh PDA plates without hygromycin. The characteristics of Ep-1PNA367R-V subcultures after contact with strain 277 were assayed for hypovirulence traits as described above (see 2.2).

## 3. Results

### 3.1. Biological Characteristics of Strain 277

**Figure 1:**
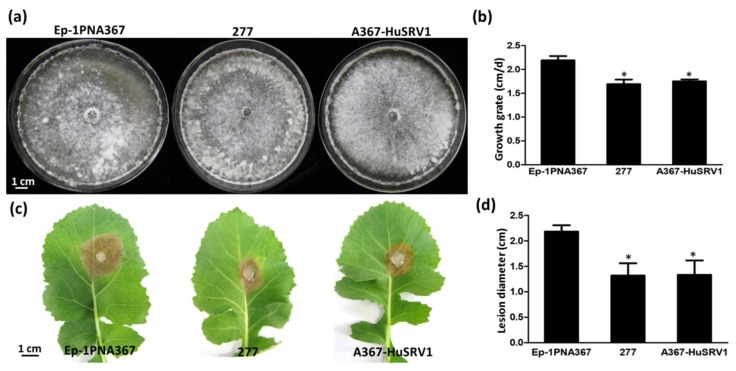
Biological characteristics of strains Ep-1PNA367 and 277, and of transfected strain A367-HuSRV1. (**a**) Colony morphology of strains Ep-1PNA367, 277 and A367-HuSRV1 grown for 7 days on PDA at 20 °C. (**b**) Growth rates of strains Ep-1PNA367, 277, and A367-HuSRV1. (**c**) Pathogenicity assay of three strains on detached rapeseed leaves at 48 hpi, 20 °C, and approximate 100% relative humidity. (**d**) Comparison of lesion diameters produced by these strains on detached rapeseed leaves. Statistical analysis was performed using one-way ANOVA followed by Dunnett’s multiple range test. Asterisks (*) on top of the bars represents significant differences when compared with Ep-1PNA367 (*p* < 0.01) based on multiple comparison analysis.

Comparisons between strains 277 and Ep-1PNA367 were conducted based on their colony morphology and virulence assay on the leaves of rapeseed. Strain 277 has similar colony morphology to strain Ep-1PNA367 on PDA (Figure 1a), but compared to the growth rate of strain Ep-1PNA367 (2.15 cm/d), strain 277 had a lower growth rate (1.65 cm/d) (Figure 1b). Moreover, strain 277 showed lower virulence as compared with strain Ep-1PNA367 (Figure 1c), and produced significantly smaller lesion (approximately 1.32 cm) in diameter than that generated by Ep-1PNA367 (2.10 cm) on the detached rapeseed leaves (Figure 1d). 

**Figure 2:**
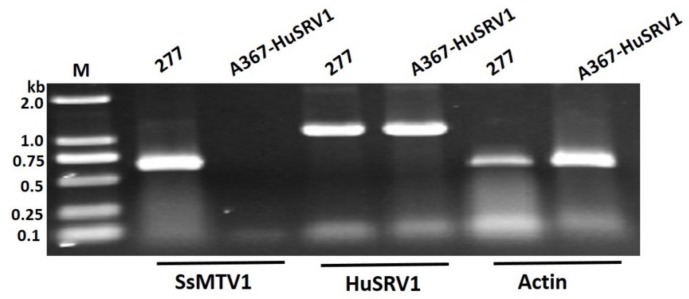
RT-PCR amplification analysis of strain 277 and transfected A367-HuSRV1. The results indicate that strain 277 harbored two viruses, while the transfected strain A367-HuSRV1 contains HuSRV1 alone. The actin gene was served as a control. RT-PCR was carried out with virus-specific primers (Table S1) for the two viruses (HuSRV1, or SsMTV1), with cDNA obtained from the total RNA extracted from strain 277 and transfected strain A367-HuSRV1. Lane M means molecular weight marker DL2000 (Takara, Dalian, China).

To confirm whether strain 277 was infected with one or more than one mycoviruses, high-throughput metatranscriptomic sequencing was conducted. Overall, Illumina sequencing generated more than 13 Gb (Giga base pairs) of raw reads, and after quality filtering, the sample contained more than 12 Gb high-quality sequence reads with a 95.47% clean ratio. Two unigene sequences (contigs 208 and 1103) were assembled after the clean transcripts were matched against the genome sequences of *S. sclerotiorum*. contigs 208 was assembled based on the 3184 reads (more than 150 nt per read), whereas contig 1103 was obtained from 15722 reads (more than 150 nt per read). Sequence analysis revealed that contigs 208 and 1103 are related to previously reported viruses, as confirmed next by RT-PCR with contig-specific primers (Table S1, Figure 2) in strain 277. Contig 208 that was 4175 bp long represented a virus related to Fusarium graminearum mycotymovirus 1 (FgMTV1) of the order *Tymovirales*, with 33% amino acid (aa) identity and 43% query coverage. This virus has approximately 90% identity to the corresponding regions of Sclerotinia sclerotiorum mycotymovirus 1 (SsMTV1/SZ-150) that was recently reported in *S. sclerotiorum* strain SZ-150 [29]. This virus, therefore, was designated as SsMTV1/277 (unpublished data). Contig 1103 (2493 bp long) was associated with the sowbane mosaic virus (SoMV) within the genus *Sobemovirus* within *Solemoviridae*, with 37% amino acid identity and 32% query coverage. This virus was tentatively designated as Hubei sclerotinia RNA virus 1 (HuSRV1). 

### 3.2. Complete Sequence and Organization of the HuSRV1 Genome

**Figure 3:**
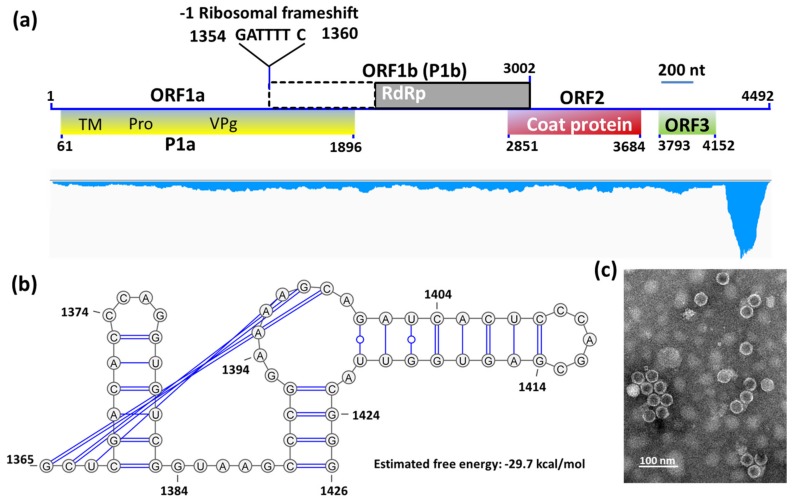
(**a**) Schematic organization and annotations of the Hubei sclerotinia RNA virus 1 (HuSRV1) genome (upper panel) and its distribution profile of transcriptomic reads (lower panel). Four putative ORFs (ORF1a, ORF1b, ORF2, and ORF3) are shown as rectangular boxes in different colors. TM, transmembrane domain; Pro, serine protease; VPg, Viral protein linked to the genome; and RdRp, RNA-dependent RNA polymerase. The dotted area in ORF1b indicates the predicted -1 ribosomal frameshifting for RdRp. The bar at the upper right of the genome structure of HuSRV1 represents the length of 200 nucleotides (200 nt). The mapped reads were more numerous in 3’-UTR than the coding regions, intergenic regions, and 5’-UTR of the genomes. (**b**) Schematic representation of -1 ribosomal frameshifting in ORF1b. The secondary structure of RNA was predicted with the KnotSeeker program. (**c**) Transmission electron microscopy examination of negatively stained purified virions of HuSRV1.

Previous reports confirm that SsMTV1-related viruses (such as FgMTV1) do not have virions [22], whereas HuSRV1-related viruses (sobemoviruses) form virions in their hosts [30]. Therefore, virions were successfully purified from the mycelia of strain 277. Transmission electron microscopy results indicated icosahedral symmetry of HuSRV1 virions, with a diameter of approximately 30 nm (Figure 3c).

The complete genome of HuSRV1 was obtained by a combination of metatranscriptomic data and RACE results. The full-length cDNA of HuSRV1 is 4492 nt long, and was predicted to have four putative ORFs, with the first three overlapping and linearly ordered (Figure 3a). The 5ˊ-untranslated region (UTR) of HuSRV1 is 60 nt long and starts with the sequence “ACAAAA”. The 3ˊ-UTR is 340 nt long and lacks the poly(A) tail structure (Table 1). The complete sequence of HuSRV1 was submitted to GenBank under the accession number MK889164.

The first ORF (ORF1a) was found to encode predicted protein P1a (612 aa) with a predicted molecular mass of 65.82 kDa, and 27% identical to the P2a protein encoded by the subterranean clover mottle virus (GenBank accession number NP_715628). Membrane protein topology prediction indicated that 23 hydrophobic residues may form a transmembrane domain at the polyprotein N-terminus; this domain involved in the formation of the putative transmembrane anchor protein (Figure S1 and Table 1). A putative transmembrane domain sequence followed by a domain with the highly conserved motif “HX_36_DX_62_TXXGXSG” of a trypsin-like serine protease (Pro) was observed in both cases. Moreover, protease-linked amino acid residues were predicted, which encodes the viral protein linked to the genome (VPg). VPg contains a highly conserved region (WAD) followed by an E/D-rich region, which was observed in ORF1a-encoded protein (Figure 3a and Table 1).

**Figure 4:**
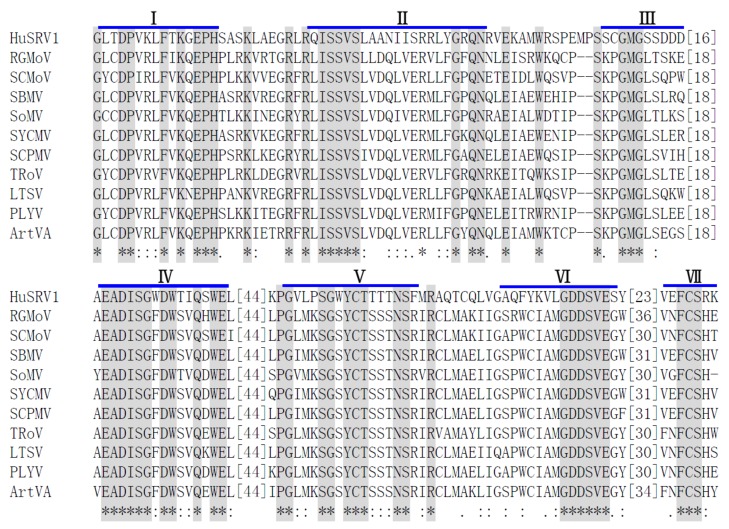
Alignment of the conserved RdRp amino acid motifs of HuSRV1 and corresponding regions in the selected viruses from *Sobemovirus*. Seven motifs (I-VII) are detected in the sequence of the conserved RdRp region. Identical residues are indicated by asterisks and highlight; conserved and semi-conserved amino acid residues are indicated by colons and dots, respectively. Numbers in square brackets correspond to the number of amino acid residues separating the motifs. Abbreviations of representative viruses of genus *Sobemovirus* are given in Table S3.

The second ORF (ORF1b) was found to overlap with ORF1a, and to encode a polyprotein P1b (592 aa) with a predicted molecular mass of 58.53 kDa. A schematic representation of the ribosomal frameshift in ORF1b is illustrated in Figure 3. The predicted shifty heptamer sequence “^1354^GATTTT^1360^” followed by a frameshift signal is present before the overlap of ORF1a and ORF1b, and an RNA pseudoknot (nt 1365–1426) with the free energy value of −29.7 kcal/mol is located downstream of the shifty heptamer motif (Figure 3a,b). A multiple sequence alignment was generated based on the amino acid sequence of the core RdRp domain of HuSRV1 and representative viruses from the genus *Sobemovirus* (Figure 4). This alignment revealed that ORF1b encoded protein contains seven conserved motifs (I-VII) including a putative “GDD” motif, the characteristic of RNA-dependent RNA polymerases (RdRp) (Table 1 and Figure 4). Therefore, ORF1b encodes a replicase of HuSRV1, and may be translated into a fusion protein with ORF1a encoded protein by a −1 ribosome frameshift mechanism.

The third ORF (ORF2) was found to overlap with the 3ˊ-end of ORF1b and encodes the viral coat protein (CP). CP contains 278 aa and has a predicted molecular weight of 30.18 kDa. The HuSRV1 CP was found to contain a shell (S) domain (Table 1). The predicted three-dimensional (3D) structure is displayed in Figure S2 (quality, 100% confidence interval).

The fourth ORF (ORF3) with the unknown function was found near the 3′-terminus of HuSRV1, and encodes a 120 aa protein with a predicted molecular mass of 12.92 kDa.

### 3.3. Evolutionary Relationship of HuSRV1

**Figure 5:**
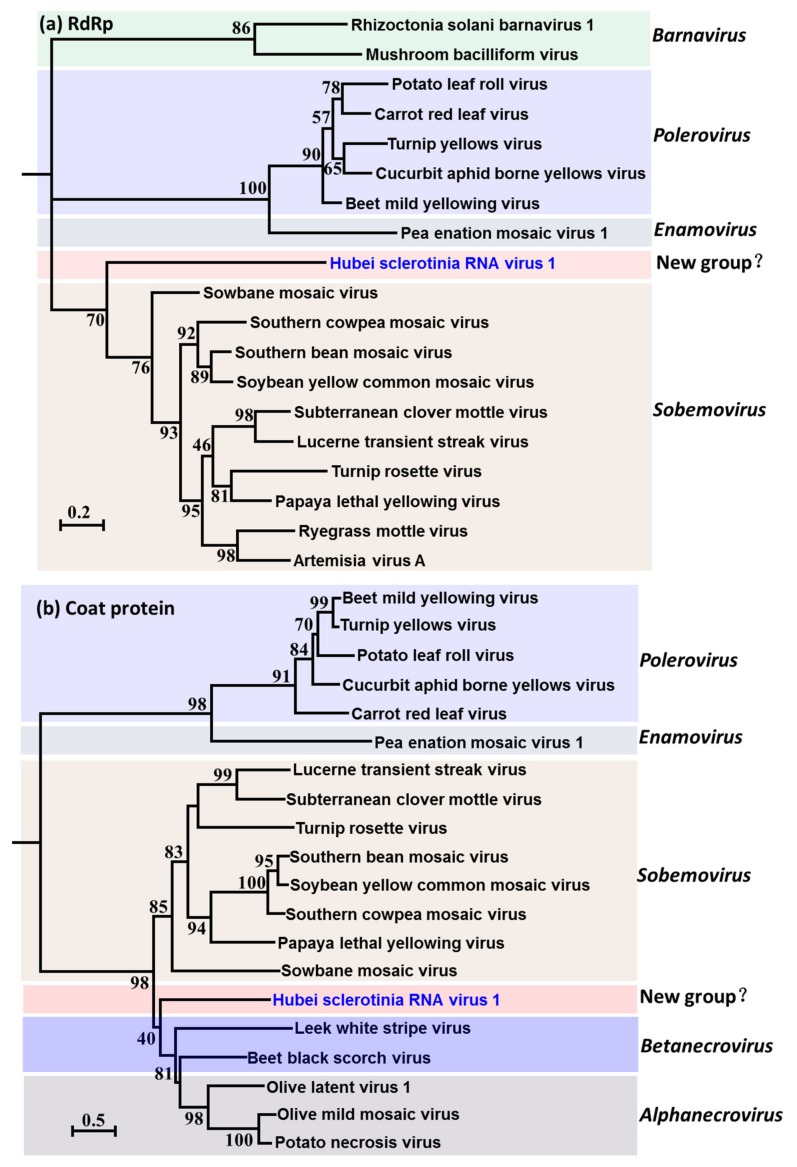
Evolutionary relationship of Hubei sclerotinia RNA virus 1 (HuSRV1). (**a**) An ML phylogenetic tree constructed based on the alignment of the aa sequences of the conserved motifs in the RdRp domain in HuSRV1 and selected viruses from the genera *Sobemovirus*, *Polerovirus*, *Enamovirus*, and *Barnavirus*. (**b**) Phylogenetic analysis of HbSRV1 and selected RNA viruses from the genera *Sobemovirus*, *Polerovirus*, *Enamovirus*, *Betanecrovirus* and *Alphanecrovirus* based on an ML tree inferred from the full sequence of the coat protein. The number on branches indicates results of analyses of 1000 bootstrap replicates, and is shown at the nodes for values greater than 50%. The bar at the lower left quarter represents the genetic distance corresponding to branch lengths. HuSRV1 reported in the present phylogenetic tree was emphasized with blue color. The accession number of viruses used for phylogenetic analysis was listed in Tables S3 and S4.

HuSRV1 evolutionary relationship analyses were based on the proteases (P1a), core RdRp, and coat protein. To generate a protease phylogram, HuSRV1 amino acid sequences and those of selected viruses from the genus *Sobemovirus* were used (Figure S3 and Table S2). The results suggested that HuSRV1 is phylogenetically related to members of the genus *Sobemovirus*, but forms a well-supported branch by itself. To generate the RdRp phylogram, the amino acid sequence of the core HuSRV1 RdRp domain and selected viruses of genera *Sobemovirus*, *Polerovirus*, *Enamovirus*, and *Barnavirus* were utilized (Figure 5a and Table S3). As expected, HuSRV1 formed an independent branch that was more closely related to sobemoviruses than poleroviruses, enamoviruses, suggesting that HuSRV1 is the first-ever sobemovirus-related virus detected in fungi (to the best of our knowledge). For the CP phylogram, the amino acid sequence of HuSRV1 CP and selected viruses of *Alphanecrovirus* and *betanecrovirus*, with an exception of viruses used in RdRp phylogenetic analysis, were employed (Figure 5b and Table S4). The phylograms indicated that HuSRV1 is more related to alphanecroviruses and betanecroviruses than to sobemoviruses, and forms a separate sub-clade in the phylogenetic tree. Thus, combining the analysis of the genomic organization and phylogenetic tree, HuSRV1 is a positive-sense, single-stranded RNA virus with unique features, and we propose the establishment of a new genus (Hbsclerovirus: Hubei sclerotinia virus) to accommodate this novel virus.

### 3.4. Transfection Assay and Biological Traits of S. sclerotiorum Infected by HuSRV1

Transfection of the virulent strain Ep-1PNA367 with purified HuSRV1 virions was achieved by the PEG-mediated method. The total RNA of the virus-transfected strain A367-HuSRV1 was extracted. Transfection was confirmed by viral RNA extraction, followed by RT-PCR amplification with virus-specific primers for either HuSRV1 or SsMTV1 (Table S1). The results suggested that strain A367-HuSRV1 was infected by HuSRV1 without SsMTV1 (Figure 2). The new *S. sclerotiorum* isolate, A367-HuSRV1, displayed debilitation phenotypes, including slower growth on PDA and less virulence on detached rapeseed leaves (Figure 1). These results directly confirmed that HuSRV1 is associated with hypovirulence in *S. sclerotiorum*.

### 3.5. Transmission Assay of the Dual Culture of Strain 277 and Ep-1PNA367R

**Figure 6:**
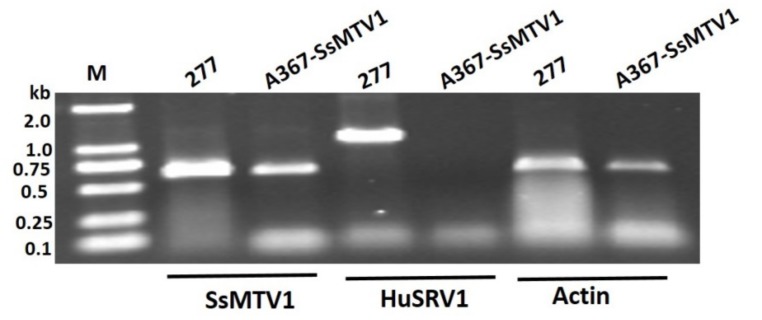
RT-PCR amplification results of the assay of transmission between virus-infected strain 277 and strain Ep-1PNA367R, with primers specific for two viruses (HuSRV1and SsMTV1). The actin gene was used as control. Lane M means molecular weight marker DL2000 (Takara, Dalian, China).

The dual-culture method was implemented for the assay of virus transmission from strain 277 to Ep-1PNA367R in the same PDA plates for seven days at 20 °C, and a clear intense black-pigmented zone (an evident antagonism line) appeared at the interface region of strains 277 and Ep-1PNA367R, suggesting that those two strains were vegetative incompatibility. Two hyphal plugs were picked from the margins of strain Ep-1PNA367R and subcultured on hygromycin-containing PDA plates. Total RNA was extracted from new strains. cDNA was synthesized from the total RNA and was subjected to PCR amplification with virus-specific primers for either HuSRV1 or SsMTV1 (Table S1). Electrophoretic analysis of RT-PCR products indicated that only SsMTV1 was transmissible to virulent strain Ep-1PNA367, and the new *S. sclerotiorum* strain A367-SsMTV1 lacks HuSRV1 (Figure 6). These results meant that HuSRV1 was not transmissible to the vegetatively incompatible *S. sclerotiorum* strain Ep-1PNA367R, on the contrary, SsMTV1 can be transmitted between the two strains in different vegetative compatibility groups.

## 4. Discussion

In this study, an ssRNA virus, HuSRV1, was detected in a hypovirulent strain 277, and its full-length genome was obtained and characterized. Sequence analysis of the HuSRV1 genome uncovered the presence of conserved domains or motifs including virus genome-linked protein (VPg), proteases, and RdRp translated via -1 ribosomal frameshifting that is the characteristic of +ssRNA plant viruses belonging to the genus *Sobemovirus* within *Solemoviridae* (https://talk.ictvonline.org/taxonomy/) [31]. 

The virions purified from hypovirulent strain 277 had the same icosahedral symmetry of capsids (Figure 3c), similarly to the virions of the genus *Sobemovirus* [30]. The molecular weight of the capsid protein was determined and turned out to be in the range of 24–30 kDa. Previous studies suggest that *Sobemovirus* CP is translated through subgenomic RNA (sgRNA) [31]. The HuSRV1 genome is 4,492 nt long and is linear. The genome of HuSRV1, like that of other sobemoviruses, has four putative ORFs (Figure 3a), but has a completely different arrangement and lacks ORFx that was recently confirmed to have a potential biological function in sobemoviruses [32].

The 5ˊ- end of the HuSRV1 genome commences with the sequence “ACAAAA”, absent from the 5’ end of the imperata yellow mottle virus [33] and the cooks foot mottle virus [34]. The motif of “CACAAAA” is present at the 5ˊ- end of genomic and subgenomic sequences of many sobemoviruses [30], while the initial C may not be present in HuSRV1. As in other sobemoviruses [30], the polypurine tract (GAGAAAAGAA) is absent in the 5ˊ-UTR of HuSRV1. The 3ˊ-UTR of HuSRV1 is 340 nt long. In the *Sobemovirus* genome, the 3ˊ-UTR shows conservation only at the marginal sequence. In other members of genus *Sobemovirus*, the first ORF (ORF1) encodes protein 1 (P1) [35,36]. Before the end of this ORF, a non-AUG mechanism (leaky scanning) forms an ORFx in sobemoviruses [32]. This leaky scanning mechanism could not be predicted in the HuSRV1 genome because of its unique genome arrangement (Figure 3a), i.e., the genome commencing with ORF1a encoding polyprotein P1a.

Analysis of P1a via BlastP uncovered a chymotrypsin-like methyltransferase domain as reported for other sobemoviruses [30]. Analysis of amino acid similarities between HuSRV1 and sobemoviruses showed 24.0%, 25.3%, and 27.6% identity with sowbane mosaic virus, sesbania mosaic virus, and southern yellow common mosaic virus, respectively. The N-terminal part of HuSRV1 manifested no considerable conservation as compared with the *Sobemovirus* polyprotein (P2a), but a transmembrane helix prediction indicated a transmembrane anchor at the N-terminus of protein P1a, followed by conserved regions of protease (Pro) and VPg (Figure 3 and Figure S1). 

The ORF1b of HuSRV1 is similar to *Sobemovirus* ORF2b. The C-terminal region of HuSRV1 ORF1b was found to contain an essential conserved motif (GDD) based on multiple sequence alignments with other members of the genus *Sobemovirus* [31]. This domain contains seven conserved motifs, which classified core RdRp motifs into superfamilies. ORF2b is probably translated as a fusion protein P1ab through -1 ribosomal frameshifting. Nevertheless, multiple sequence alignment failed to detect the conserved slippery sequence “UUUAAAC” upstream of ORF1b followed by a stem-loop structure as observed in sobemoviruses [30,37]. Aside from this difference, HuSRV1 has the slippery sequence “GATTTT” that was detected in alphamesoniviruses (Family: *Mesonivaridae*; Genus: *Mesonivirus*) [38], implying -1 ribosomal frameshifting was involved in translation in HuSRV1 (Figure 3a,b).

HuSRV1 ORF2 encodes CP, which corresponds to the fourth ORF in the genome arrangement of sobemoviruses. In most of sobemoviruses, CP is translated from a conserved “ACAAA” motif located upstream of the translation start site of CP, at the 5ˊ-terminus of sgRNA [30], but this motif is absent in CP of HuSRV1. HuSRV1 CP contains only an S domain, in contrast to other sobemoviruses that generally have an N-terminal R domain and the virion core building block S domain at the C-terminus [30]. The invariant CP amino acid residues of HuSRV1 manifested conservation based on multiple sequence alignment with other sobemoviruses (Table 1).

Four of these amino acid residues are involved in Ca^2+^ binding, and others are likely to be involved in backbone conformation [39]. A predicted 3D structure of HuSRV1 CP indicated that three CP chains (A, B, and C) are capable of bonding with Ca^2+^ binding sites (Figure S2). Hence, this novel HuSRV1 is different in the genome arrangement from known sobemoviruses. The function of its ORF3 remains unknown, and this contains no conserved domain according to BlastP analysis. HuSRV1 has similar properties to those of previously reported viruses of the genus *Sobemovirus*, but unique features such as ribosomal frameshifting and CP domains set the new virus apart. Phylogenetic analyses confirmed that HuSRV1 is related to sobemoviruses, albeit forming a sub-clade separate from all other known plant viruses in a different genus in phylograms. Thus, we proposed to establish a new genus (Hbsclerovirus: Hubei sclerotinia virus) to accommodate novel virus HuSRV1 and its related viruses that may be discovered in the near future.

It is natural and not unusual to detect co-infection with multiple mycoviruses in a single fungal strain. Twelve dsRNA segments are shown to make up the genome of mitovirus co-infected a strain of *Ohiostoma novo-ulmi* [40]. There are many examples of mixed infections including hypovirulent strains of *S. sclerotiorum* (SZ-150, 16235, and Ep-1PN), with two or three other mycoviruses reported to cause co-infection in single hypovirulent strain [18,23,29]. In this study, deep sequencing data revealed that strain 277 is co-infected with two different mycoviruses, which provides another example of the complexity of mycoviruses affecting the plant fungus *S. sclerotiorum*.

Mycoviruses associated hypovirulence could not be effectively used for controlling fungal diseases because of the key limiting factor of vegetative incompatibility of fungal hosts [5]. In this regard, SsPV1 and SsHADV-1 have shown the potential to overcome vegetative incompatibility, and SsPV1 also holds promise for transmission to incompatible fungal genetic groups because these mycoviruses can overcome the vegetative incompatibility of their host [19,20]. In this study, HuSRV1 could not be horizontally transmitted to a given vegetatively incompatible strain via hyphal anastomosis. On the contrary, SsMTV1 that does not form virion can be transmitted. This requires further characterization of how such transmission happens, and the effect of A367-SsMTV1 on the growth rate and virulence requires further characterization too.

In summary, HuSRV1 is a novel virus that is related to, but different from known viruses, in terms of host and genomic characteristics. Fungi are the host of HuSRV1, while plants are the host of sobemoviruses. It’s reasonable to speculate that HuSRV1 can infect plants as well based on the phylogenetic analysis. The number of ORF in the HuSRV1 genome is the same as that in the *Sobemovirus*, but with a completely different arrangement. Our evolutionary analyses underscore the need to establish a new genus (Hbsclerovirus) to accommodate this new virus. 

## Figures and Tables

**Table 1:** Nucleic acid and amino acid sequence motifs identified in the genome of HuSRV1 based on the previously reported members in genus *Sobemovirus* [30].

**Nucleotide Sequence motifs**		
**Motif**	**Nucleotide positions**	**Description**
ACAAAA	1–6	Precedes the P1a start codon
GATTTTC	1354–1360	−1 ribosomal frameshifting signal
**Amino acid (AA) sequence motifs**		
**Motif**	**AA positions**	**Description**
Transmembrane helix	21–43 (P1a)	Predicted transmembrane domain
HX_36_DX_62_TXXGXSG	186–292 (P1a)	Pro: Serine protease
WADLDDEDEDX_3_DD	393–407 (P1a)	VPg: ED-rich region
RdRp motifs I-VII	I: 241–252 II: 265–290 III: 301–309 IV: 330–342 V: 387–411 VI: 417–428 VII: 455–464	Motifs that classify *RdRp* genes into superfamilies
GX_3_TX_3_NX_19_GDD	395–425 (RdRp)	Conserved region
S (shell) domain	43–272 (CP)	Shell domain involved in subunit interactions
P_144_, G_152_, D_161_, D_164_, P_239_, G_257_, N_276_	144–276 (CP)	Conserved invariant amino acid residues
D_161_, D_164_, T_220_, N_276_	161–276 (CP)	Ca^2+^ binding sites

## Supplementary Materials

The following are available online at https://www.mdpi.com/1999-4915/11/8/759/s1. Figure S1. The predicted transmembrane helix domain of polyprotein P1a (according to TMHMM server 2.0). Figure S2. Two-sided view of the predicted 3D structure of CP of HuSRV1 (Phyre2). Each chain of CP is represented by a different color: Chain A, red; Chain B, green; and Chain C, blue. Small light green beads indicate the position of Ca^2+^ binding sites (UCSF Chimera). Figure S3. Evolutionary relationship of the protease of Hubei sclerotinia RNA virus 1 (highlighted in blue) with other viruses of the genera *Sobemovirus*, *Polerovirus*, *Enamovirus*, and *Barnavirus*. The number of branches indicates the results of analyses of 1000 bootstrap replicates. The bar at the lower left quarter represents the genetic distance. The accession number of viruses used for phylogenetic analysis was listed in Table S2. Table S1. The list of the primers used in the study. Table S2. The viral sequences selected for the phylogenetic analysis of protease domains. Table S3. The viral sequences selected for the phylogenetic analysis of core RdRp domains. Table S4. The viral sequences selected for the phylogenetic analysis of coat proteins
