# Supplementary material for: A Novel RNA Virus Related to Sobemoviruses Confers Hypovirulence on the Phytopathogenic Fungus Sclerotinia sclerotiorum
Source: Viruses. 2019 Aug 16;11(8):759. doi: 10.3390/v11080759 (PMC6722724; doi:10.3390/v11080759)
Supplement: Supplementary file 1 [file viruses-11-00759-s001.pdf]

viruses
MDPI

Supplementary materials

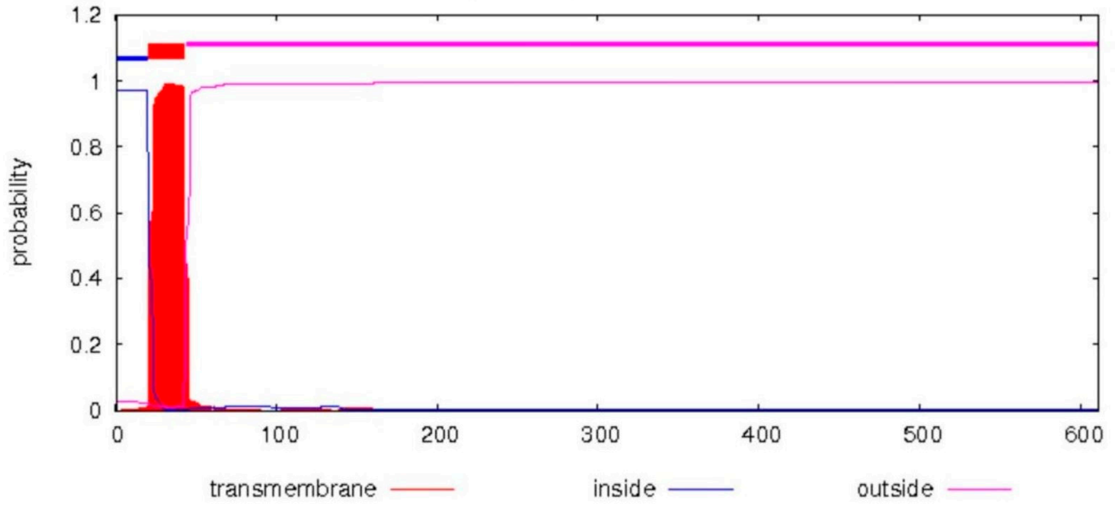

Figure S1. The predicted transmembrane helix domain of polyprotein P1a (according to TMHMM server 2.0).

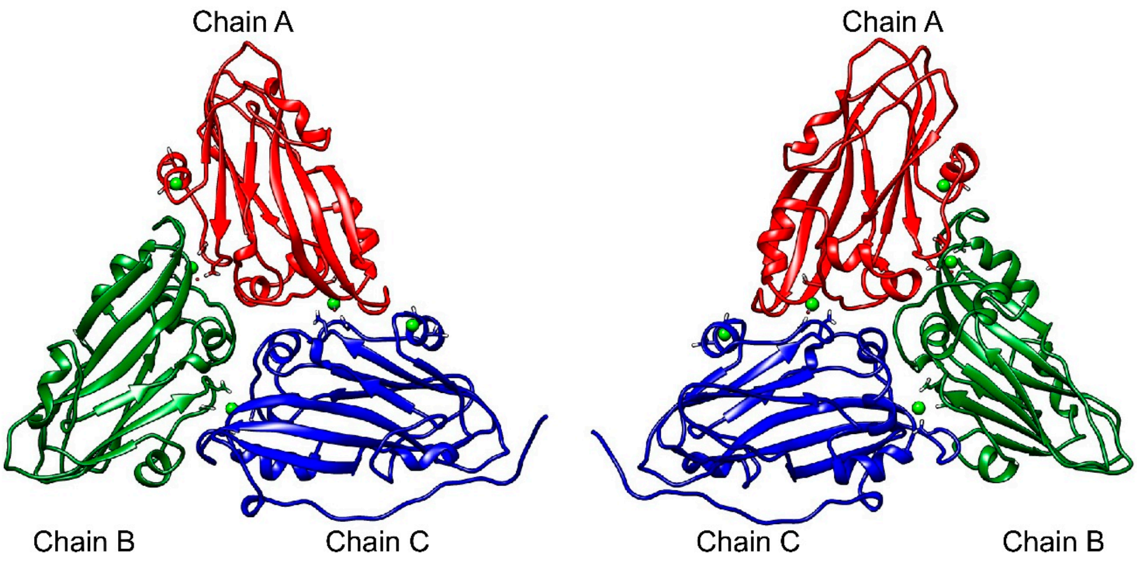

Figure S2. Two-sided view of the predicted 3D structure of CP of HuSRV1 (Phyre2). Each chain of CP is represented by a different color: Chain A, red; Chain B, green; and Chain C, blue. Small light green beads indicate the position of Ca^{2+} binding sites (UCSF Chimera).

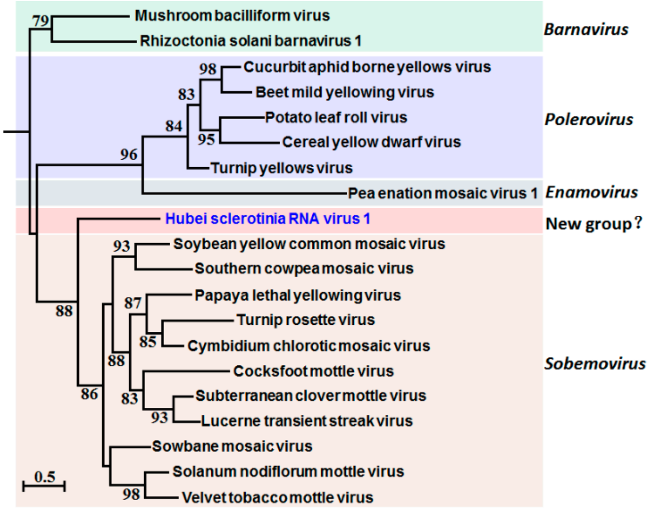

Figure S3. Evolutionary relationship of the protease of Hubei sclerotinia RNA virus 1 (highlighted in blue) with other viruses of the genera *Sobemovirus*, *Polerovirus*, *Enamovirus*, and *Barnavirus*. The number on branches indicates results of analyses of 1000 bootstrap replicates. The bar at lower left quarter represents the genetic distance. Accession number of viruses used for phylogenetic analysis was listed in Table S2.

Table S1. The list of the primers used in the study.

Primer Name	Sequence
HuSRV1-F1	5′-CTGCTGTCGGTTCTTCAAC-3'
HuSRV1-R1	5′-ACATTCAGCCTACCAGACG-3'
HuSRV1-F2	5′-CTGCTGTCGGTTCTTCAAC-3'
HuSRV1-R2	5′-ACATTCAGCCTACCAGACG-3'
RACE-3RT/ Random dN6 primer	5'-CGATCGATCATGATGCAATGCNNNNNN-3'
PC3-T7 Loop adapter	5′pGGATCCCGGGAATTCGGTAATACGACTCACTATATTTTTATA GTGAGTCGTATTA-OH-3´
PC2 primer	5′-p-CCGAATTCCCGGGATCC-3′
Seq. specific primer - 5end HuSRV1-5end-R1	5′-AGTGCTTTGACACCGAGAAC-3′
Seq. specific primer - 5end HuSRV1-5end-R2	5′-GCCAGTACCAATCGAAAGAG-3′
Seq. specificprimer - 3end HuSRV1-3end-F1	5′-CCGCAACAACACTGTCACTGG-3′
Seq. specific primer -3end HuSRV1-3end-F2	5′-GGTAATGCTAGCGGCAACACTG-3′
SsMTV1-F	5′-CGAGCCATTCCCCGATACA-3′
SsMTV1-R	5′-GAGTCCTGACCAATGAGTGCTG-3′

Table S2. The viral sequences selected for the phylogenetic analysis of protease domains.

Virus family	Genus	Virus name	Abbreviation	Genbank accession no. of Protease
Uassigned	*Sobemovirus*	Turnip rosette virus	TRoV	AAO24318.2
		Artemisia virus A	ArtVA	YP_006331062.1
		Southern bean mosaic virus	SBMV	YP_007438857.2
		Velvet tobacco mottle virus	VTMoV	AEE36644.1
		Lucerne transient streak virus	LTSV	YP_007438850.1
		Papaya lethal yellowing virus	PLYV	YP_006589926.1
		Rice yellow mottle virus	RYMV	YP_007438861.1
		Ryegrass mottle virus	RGMoV	YP_007438854.1
		Sesbania mosaic virus	SeMV	NP_066392.4
		Solanum nodiflorum mottle virus	SNMV	YP_009344992.1
		Southern cowpea mosaic virus	SCPMV	NP_042301.2
		Sowbane mosaic virus	SoMV	YP_002158814.1
		Soybean yellow common mosaic virus	SYCMV	YP_004869650.1
		Subterranean clover mottle virus	SCMoV	NP_715628.1

Table S3. The viral sequences selected for the phylogenetic analysis of core RdRp domains.

Virus Family	Genus	Virus name	Abbreviation	Genbank accession no. of RdRp
Unassigned	*Sobemovirus*	Southern bean mosaic virus	SBMV	YP_007438858.2
		Soybean yellow common mosaic virus	SYCMV	BBB03266.1
		Southern cowpea mosaic virus	SCPMV	APA23084.1
		Papaya lethal yellowing virus	PLYV	AFP67700.1
		Sowbane mosaic virus	SoMV	ADD64689.1
		Lucerne transient streak virus	LTSV	YP_007438849.1
		Ryegrass mottle virus	RGMoV	YP_007438853.1
		Artemisia virus	ArtVA	YP_006331061.2
		Turnip rosette virus A	TRoV	YP_008869286.1
Barnaviridae	*Barnavirus*	Mushroom bacilliform virus	MBV	AAA53090.1
		Rhizoctonia solani barnavirus 1	RsBarV1	ALD89112.1
Luteoviridae	*Enamovirus*	Pea enation mosaic virus 1	PEMV-1	AAA72297.1
Luteoviridae	*Polerovirus*	Potato leaf roll virus	PLRV	ARS33717
		Cucurbit aphid-borne yellows virus	CABYV	ALJ83663
		Beet mild yellowing virus	BMYV	AAZ57426
		Carrot red leaf virus	CtRLV	AAU04781.1
		Turnip yellows virus	TuYV	ALL26142.1
	Luteovirus	Bean leafroll virus	BLRV	AAL66234.1
		Barley yellow dwarf virus	BYDV	CAA30498.1

Table S4. The viral sequences selected for the phylogenetic analysis of Coat proteins.

Virus family	Genus	Virus name	Abbreviation	Genbank accession no. of CP
Unassigned	*Sobemovirus*	Southern bean mosaic virus	SBMV	ABH07427.1
		Sowbane mosaic virus	SoMV	YP_002158815
		Rice yellow mottle virus	RYMV	AE125520
		Cooks foot mottle virus	CfMV	NP_941377.1
		Soybean yellow common mosaic virus	SYCMV	ALR34994.1
		Southern cowpea mosaic virus	SCPMV	APA23085.1
		Turnip rosette virus	TRoV	AGP50168
		Imperata yellow mottle virus	IYMV	YP_002308437
		Sesbenia mosaic virus	SeMV	NP_066394.1
		Subterranean clover mottle virus	SCMoV	NP_715629.1
		Lucerne transient streak virus	LTSV	YP_007438851.1
Tombusviridae	*Alphanecrovi-rus*	Potao necrosis virus	PoNV	YP_009246412.1
		Olive latent virus	OLV-1	AHE40771.1
		Olive mild mosaic virus	OMMV	AIR95701.1
		Tobacco necrosis virus D	TNV-D	AIR95713.1
